# Penicillin V prophylaxis uptake among children living with sickle cell disease in a specialist sickle cell clinic in Ghana: A cross‐sectional study

**DOI:** 10.1002/hsr2.953

**Published:** 2022-11-24

**Authors:** Samuel F. Odoom, Sam K. Newton, Emmanuel K. Nakua, Kennedy G. Boahen, Samuel B. Nguah, Daniel Ansong, Isaac Nyanor, Evans X. Amuzu, Ernest Amanor, Francis A. Osei, Aliyu Mohammed, Nicholas K. Mensah, Charles Martyn‐Dickens, Alex Osei‐Akoto, Vivian Paintsil

**Affiliations:** ^1^ Department of Epidemiology and Biostatistics, School of Public Health Kwame Nkrumah University of Science and Technology Kumasi Ghana; ^2^ Child Health Directorate Komfo Anokye Teaching Hospital Kumasi Ghana; ^3^ School of Public Health Kwame Nkrumah University of Science and Technology Kumasi Ghana; ^4^ Department of Clinical Microbiology, School of Medicine and Dentistry Kwame Nkrumah University of Science and Technology Kumasi Ghana; ^5^ Department of Biochemistry and Biotechnology, College of Science Kwame Nkrumah University of Science and Technology Kumasi Ghana; ^6^ Public Health Unit Komfo Anokye Teaching Hospital Kumasi Ghana

**Keywords:** penicillin V prophylaxis, penicillin V prophylaxis adherence, sickle cell disease (SCD), Sickle Pan‐African Research Consortium (SPARCo), urine assay method

## Abstract

**Background and Aims:**

Penicillin V prophylaxis protects children living with sickle cell disease (SCD) from bacteria infections especially *Streptococcus pneumonia*. However, the uptake of penicillin V prophylaxis is difficult to assess and often poor among SCD patients. Therefore, this study sought to investigate oral penicillin V prophylaxis adherence among SCD children using urine assay and self‐reported methods and the associated factors.

**Methods:**

The study employed an analytical cross‐sectional design in the assessment of penicillin V prophylaxis adherence using both urine assay and self‐reported methods. Multiple logistic regression analysis was used to determine the factors associated with penicillin V prophylaxis adherence. A *p* value < 0.05 was considered statistically significant.

**Results:**

Among the 421 SCD patients recruited, penicillin V prophylaxis adherence was observed to be 30.0% and 68.0% for the objective and subjective methods of assessment, respectively. For the objective method of assessment, being cared for by grandparents increased the odds of penicillin V adherence (adjusted odds ratio [aOR] = 3.68, confidence interval [CI] = 1.03–13.15). However, SCD patients within the ages of 10–14 years (aOR = 0.36, CI = 0.17–0.80), >14 years (aOR = 0.17, CI = 0.05–0.61), SCD patient cared for by married caregivers/parents (aOR = 0.32, CI = 0.14–0.72), SCD patient cared for by divorced caregivers/parents (aOR = 0.23, CI = 0.07–0.75), SCD patients taking homemade (herbal) preparations for the treatment of SCD (aOR = 0.42, CI = 0.21–0.83), and inappropriate intake of penicillin V prophylaxis (aOR = 0.27, CI = 0.11–0.67) reduced the odds of penicillin V adherence. For the subjective method of assessment, taking homemade preparation (herbal) for the treatment of SCD (aOR = 0.52, CI = 0.30–0.89) and inappropriate intake of penicillin V (aOR = 0.32, CI = 0.17–0.60) reduced the odds of penicillin V adherence.

**Conclusion:**

This study reports a relatively low adherence rate of penicillin V prophylaxis among children living with SCD. Educating and counseling both SCD patients and/or caregivers on the need to be adherent to penicillin V prophylaxis could prevent complications that may arise from nonadherence.

## 1 BACKGROUND

Sickle cell disease (SCD) is the most common inherited disorder among hemoglobinopathies and is designated a global health problem.^1^, ^2^ SCD is a recessively inherited disorder predominantly distributed with descent from African, Middle Eastern, Mediterranean, Indian, South and Central American, and Asian populations.

Annually, over 300,000 children are born with this disorder globally and this number is expected to increase to 400,000 by 2050.^3^, ^4^ In sub‐Saharan Africa, about 8 in 10 children are born of SCD which is reported to be associated with a high mortality rate in such children.^5^, ^6^ In Ghana about 2 out of 100 newborns are diagnosed as having SCD with approximately 6 in 10 having the homozygous form (SS).^7^ The current daily treatment regimen such as folic acid, hematinics, penicillin V and hydroxyurea has significantly improved the health outcomes of SCD patients as well as reduced the mortality rate of the disease. In sub‐Saharan Africa, infection alone contributes to about 50.0% mortality rate among children (under 5) diagnosed with SCD.^8^, ^9^, ^10^ Penicillin V protects SCD patients from bacteria infections including *Streptococcus pneumonia*.^11^, ^12^ It has been reported that oral penicillin V prophylaxis reduces 84% pneumococcal infections and related deaths when given twice daily.^13^ Nonetheless, without penicillin V treatment, infection predisposed SCD patients to other events such as acute chest syndrome (ACS), pain, sequestration, and hyperhemolytic episodes.^14^

Although the life expectancy of SCD patients has improved in recent times, the current medication regimen is a major barrier to medication adherence. Medication adherence is a complex problem that involves the patient, caregiver, and healthcare practitioner.^15^ Adherence is defined as the extent to which patients’ actions coincide with the agreement of recommendations by healthcare providers.^16^ Medication adherence among children suffering from chronic conditions is problematic.^17^ Averagely about 50% of children with chronic conditions are adherent to medication with wide varying levels ranging from 11% to 93%^18^ which decreases with increasing age to about 5% to 15%.^17^ Given the benefits of penicillin V prophylaxis in reducing infection‐related complications in SCD patients, it is imperative to effectively monitor penicillin V adherence among SCD patients. However, the existing body of literature on penicillin V adherence among SCD patients is limited and not contemporary to inform policies for present issues.^19^, ^20^, ^21^ A literature search suggested that in Ghana and sub‐Saharan Africa, the level of adherence to penicillin V prophylaxis and factors influencing adherence has not been well studied and, therefore, called for an investigation.

The current study assumed that adherence to penicillin V medication is poor among SCD children and this is because children are vulnerable and their medication adherence is significantly influenced by their parents. The present study, therefore, sought to determine oral penicillin V prophylaxis adherence among SCD children and the associated factors. To the best of our knowledge, this is the first study assessing the adherence to penicillin V prophylaxis among children living with SCD in Ghana using both self‐reported and urine assay methods of assessment. The findings from this study will provide an empirical understanding on how children with SCD in Ghana take oral penicillin V prophylaxis.

## 2 METHODOLOGY

### 2.1 Study design

The study employed an analytical cross‐sectional design that involved both quantitative data collection techniques and laboratory analysis of urine samples of SCD children who presented at the outpatient clinic from October 1, 2021, to January 31, 2022.

### 2.2 Study area

The study was conducted at Komfo Anokye Teaching Hospital (KATH), Paediatric SCD Clinic. KATH is located in the Kumasi metropolis, the regional capital of the Ashanti Region. The Ashanti Region is the second largest of the 16 administrative regions of Ghana with a population of 5.4 million.^22^ Due to its central location in Ghana, it is accessible from all corners of the country.^23^ KATH is a tertiary hospital with a bed capacity of 1200 and serves as a major referral center for the middle and northern zones of Ghana.^24^ The Child Health Directorate of KATH runs a 24‐h specialist outpatient clinic and six in‐patient wards. The SCD outpatient clinic is one of the specialist clinics organized by the directorate and runs 4 days a week and its being run by four specialist pediatricians, three resident's doctors, and six nurses. Averagely, the clinic sees about 500 SCD patients monthly. Based on the standard operating procedure for the management of SCD at the clinic, SCD patients below 3 years and those 3 years and above are scheduled for a visit every 2 and 3 months, respectively. Also, routine laboratory tests such as complete blood counts with reticulocyte counts, kidney function test, liver function test, and eye examination are done.^25^ In terms of routine regimen, SCD patients are given folic acid, hematinic, hydroxyurea (SS genotype and some SC genotype with severe form of crisis), and penicillin V (being prescribed to all age groups).

### 2.3 Study population

Children between 2 and 17 years diagnosed with SCD who were in steady‐state (Steady state was defined as any SCD patients free from infection, painful episodes or other disease processes) and registered into the Kumasi Sickle Pan African Research Consortium (SPARCo) registry were included in the study. However, SCD patients who had consumed any other antibiotics 72 h before recruitment and had sought healthcare at the clinic for less than 12 months were excluded.

## 3 SAMPLING

### 3.1 Sample size estimation

The sample size was calculated using Cochran's prevalence formulae.^26^ A previous study conducted in New York at Children's Hospital of Buffalo, reported penicillin V adherence level of 43.1%.^27^ Using a 95% confidence interval, 5% error margin and accounting for a nonresponse of 10%, a minimum sample size of 410 was estimated.

### 3.2 Sampling techniques

**Figure 1:**
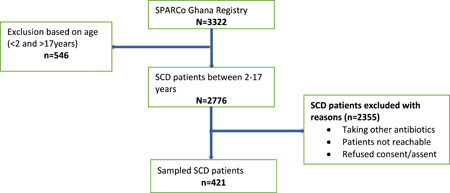
Flow chart of Sampling of SCD patients. SCD, sickle cell disease.

Simple random sampling was employed to recruit the estimated sample from the SCD patients from the SPARCo‐Kumasi Registry.^28^ With ethical approval, the SCD patients within the stated age categories were sampled from the registry using the research randomizer online^29^ taking into consideration the study sample size. The sampled patients were contacted during their clinic visit at the paediatric sickle cell clinic at KATH. Instances where sampled patients were excluded, resampling was done to replace the excluded patients until achieving the required sample size (Figure 1).

### 3.3 Study procedure

The sampled SCD patients were screened for eligibility. Consent was obtained from parents or legal guardians of all patients with assent obtained from patients 7 years and above. Urine sample containers were given to the patients to provide a urine sample. The procedure for the collection of the urine sample was explained and demonstrated to the patients and/or the caregivers to provide a 5 ml clean catch urine.^30^ The samples were refrigerated at 4°C immediately upon receipt. A 30 min questionnaire was then administered by the research assistants to the caregiver and study participant. The urine sample was then sent to the laboratory for the detection of penicillin V in the urine.

### 3.4 *Micrococcus luteus* method for detection of penicillin V in patient urine sample

According to Groove and Randall, penicillin V can be detected in urine as low as 0.005 unit/mm and also be detectable 16 h after last dose of consumption.^31^ The indicator organism for the detection of the penicillin V was *M. luteus. M. luteus* is a member of the family Micrococcaceae and is usually regarded as contaminants from skin and mucus membrane which forms yellowish colonies and appears as a gram‐positive coccus typically arranged in tetrads.^32^ The following technique was used in the detection of penicillin V in the urine sample of SCD patients.

A 25 mm sterile square filter paper was dipped/placed in a 200 µl urine sample to be tested. The filter paper was placed on blood agar (5% sheep blood) that has been completely streaked with 105 cfu/ml of *M. luteus* (indicator organism). This was incubated for at least 18 h aerobically at 37°C. Zones of inhibition of any size around the filter paper were considered as a presence of penicillin V. The technique has several advantages.

- Contamination of the urine sample and/or the filter papers does not affect the zone of inhibition of the *M. luteus*.
- Also, the urine can be left unrefrigerated for at least 12 h or refrigerated for at least 48 h and if penicillin V is present in the urine, it was still evident. However, all samples were not tested beyond this time interval. This was confirmed from some sampled samples.

**Figure 2:**
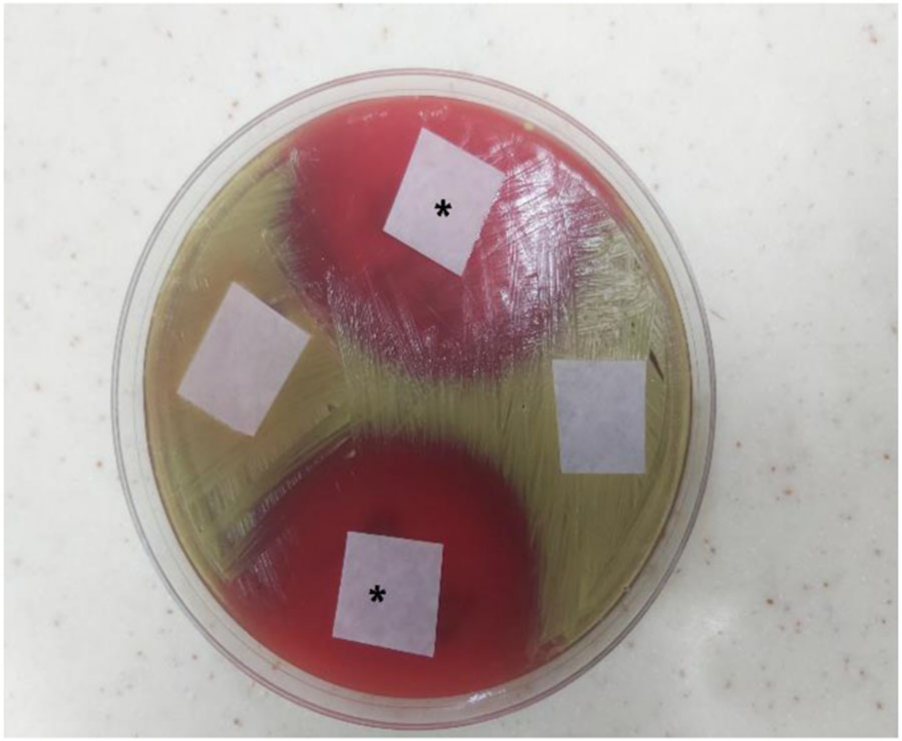
^*^Zone of Inhibition of *Micrococcus luteus* around the filter paper

A typical agar plate with evidence and nonevidence of zone of inhibition of bacteria growth is shown in Figure 2.

## 4 DATA COLLECTION METHODS AND INSTRUMENT

The tool for the study was pilot‐tested at the Kwame Nkrumah University of Science and Technology noncommunicable disease clinic (the clinic also sees paediatric SCD patients) and Maternal and Child Health Hospital, all in Kumasi. Forty‐one participants, making 10% of the calculated sample size was used for the pilot test, to accomplish a reasonable power to ensure the reliability and validity of the questionnaire. For consistency, the questionnaire was translated into the local language (Asante Twi). The structured questionnaire was answered by the SCD patients and/or caregivers (the questionnaire was answered by SCD patients (≥8 years) and their caregivers, and for SCD patients below 8 years, their caregivers answered the questionnaire on their behalf). The study instrument consisted of background characteristics of caregivers and patients, clinical history, and barriers to penicillin V consumption. These data were collected using an electronic version of the questionnaire designed with Research Electronic Data Capture (REDCap).^33^ These data were routinely reviewed to ensure completeness and accuracy.

## 5 MEASUREMENT AND STATISTICAL ANALYSIS

### 5.1 Measurement

#### 5.1.1 Dependent/outcome variable

The outcome variable, “penicillin V adherence” was measured in two forms, the urine assay (objective) method being the primary outcome variable and the self‐reported (subjective) being the secondary outcome variable. The primary outcome variable was measured as binary “adherent” and “nonadherent.” Indication of any evidence of any zone of inhibition of the *M. luteus* on the agar plate was termed as “adherent” and “nonadherent” indicated its absence. Self‐reported adherence was measured by asking the patient or caregiver who reported taking at least one dose of penicillin V within 15 h of recruitment. Patients who reported to have consumed penicillin V within 15 h were deemed “adherent” and above 15 h were considered “nonadherent.” Patients who had consumed penicillin V within 15 h are more likely to have penicillin V present in their urine since penicillin V can still be detectable 16 h after the last dose.^34^ Adherent and nonadherent were coded as 1 and 0, respectively.

### 5.2 Independent/predictor variables

The predictor variables that were measured in this study were background characteristics of SCD patients and caregivers, clinical history of SCD patients, and barriers to penicillin V adherence (the responses to the barriers to adherence were “Yes” and “No.” Scores were generated for each “Yes” (1) and “No” (0) response). Intake of penicillin V was categorized as inappropriate or appropriate. Inappropriate intake was defined as SCD patients who underutilized (underuse) and/or overutilized (overuse) penicillin V medication respective of age.

#### 5.2.1 Statistical analysis

Data cleaning and analysis were done using Stata (STATA/SE version 17.0). Descriptive statistics were performed for all variables and expressed as mean and standard deviation for continuous variables. Categorical variables were expressed as proportions and presented using tables and charts. *χ* ^2^ test of association was used to compare the objective and subjective assessments.

Multiple logistic regression analysis was used to determine the possible factors associated with both subjective and objective methods of penicillin V adherence and also to measure the strength of the associations between the independent variables and penicillin V adherence. The results were reported as odds ratios (ORs) and 95% CIs around the respective ORs. The final model was built using the stepwise selection of independent variables (factors) method with a *p* < 0.05 significance level. The Hosmer–Lemeshow goodness‐of‐fit test was used to validate the model.

## 6 RESULTS

### 6.1 Background characteristics of SCD patients

A total of 421 SCD patients were recruited, with more than half 224 (53.21%) being males. The age of the SCD patients ranged from 2 to 17 years with a mean of 9.25 (±4.00) years. The highest number 163 (38.72%) of patients were in the 5–9 years group. Patients with Hemoglobin SS (HbSS) constituted the majority with 331 (78.62%). Most primary caregivers 377 (89.55%) were the biological parents of the patients (Table 1).

**Table 1:** Background characteristics of sickle cell disease patients

Variable	Frequency (*n* = 421)	Percentage
Gender		
Female	197	46.79
Male	224	53.21
Age (years)		
<5	58	13.78
5–9	163	38.72
10–14	156	37.05
>14	44	10.45
Mean (SD)	9.25 (±4.00)	
SCD phenotype		
HbSC	90	21.38
HbSS	331	78.62
Residential status		
Rural	81	19.24
Suburban	204	48.46
Urban	136	32.30
Schooling status		
Not schooling	11	2.61
Schooling	410	97.39
Level of education	*(n=410)*	
Preschool	121	29.51
Basic education	273	66.59
Secondary education	16	3.90
Primary caregiver		
Biological parent	377	89.55
Grandparent	23	5.46
Other	21	4.99

Abbreviations: HbSC, hemoglobin SC; HbSS, hemoglobin SS; SCD, sickle cell disease; SD, standard deviation.

### 6.2 Background characteristics of primary caregivers

Hundred and eighty caregivers/parents (42.76%) were between the ages of 38–47 years and the majority 312 (74.11%) were married. The highest educational level attained by most of the caregivers (234; 55.58%) was basic education. About three‐quarters of them 310 (73.63%) were self‐employed (Table 2).

**Table 2:** Background characteristics of primary caregivers

Variable	Frequency (*n* = 421)	Percentage
Age (years)		
<28	20	4.75
28–37	121	28.74
38–47	180	42.76
>47	100	23.75
Median (IQR)	40 (35–47)	
Marital status		
Single	46	10.93
Married	312	74.11
Divorced	35	8.31
Widowed	28	6.65
Family size		
Mean (SD)	5.18 (±1.87)	
Educational level		
No formal education	26	6.18
Basic education	234	55.58
Secondary education	86	20.43
Tertiary education	75	17.81
Employment status		
Unemployed	44	10.45
Self‐employed	310	73.63
Formally employed	67	15.91

Abbreviations: IQR, interquartile range; SD, standard deviation.

### 6.3 Clinical history of SCD patients

Most 248 (58.91%) of the patients were diagnosed via the Newborn Screening Program (NSP). On average, the SCD patients assessed in this study had sought care at the outpatient clinic for 5 years [5.73 (±4.46)]. Comorbidity was observed in 20 (4.75%) of the patients. More than 141 (30.00%) had a family history of SCD with more than 10% (*n* = 72) indicating their sibling(s) were also affected. More than 89 (20.00%) reported having taken homemade medication for the treatment of their condition. Intake of penicillin V was inappropriate among 56 (13.30%) of the patients (Table 3).

**Table 3:** Clinical history of SCD patients

Variable	Frequency (*n* = 421)	Percentage
Mode of diagnosis		
NSP	248	58.91
Non‐NSP	173	41.09
Age at diagnosis (non‐NSP)	(*n* = 173)	
Mean (SD)	3.94 (±2.89)	
Duration of accessing care at KATH SCD clinic *(years)*		
≤5	233	55.34
6–10	111	26.37
>10	77	18.29
Mean (SD)	5.73 (±4.46)	
Comorbidity		
No	401	95.25
Yes	20	4.75
Family history of SCD		
No	280	66.51
Yes	141	33.49
Relative living with SCD	(*n* = 141)	
Mother	22	5.23
Father	12	2.85
Auntie/uncle	23	5.46
Sibling(s)	72	17.10
Grandparent	11	2.61
Taking homemade medication for SCD		
No	332	78.86
Yes	89	21.14
Intake of penicillin V		
Inappropriate	56	13.30
Appropriate	365	86.70

Abbreviations: Non‐NSP, non‐Newborn Screening Program; NSP; Newborn Screening Program; SCD, sickle cell disease; SD, standard deviation.

### 6.4 Method of assessment of adherence of penicillin V prophylaxis

Self‐reported adherence was observed in 288 (68.41%) whilst the urine assay (objective) revealed 125 (30.0%) of the SCD patients being adherent to penicillin V. Statistically significant association was observed between the urine assay (objective) method and self‐reported method of adherence (*p* < 0.001) (Table 4).

**Table 4:** Urine assay and self‐reported method of adherence level of penicillin V prophylaxis

Variable	Urine assay method		Total	*p* Value
Self‐reported method	Nonadherent, *n* (%)	Adherent, *n* (%)		
Nonadherent	115 (86.47)	18 (13.53)	133 (31.59)	<0.001
Adherent	181 (62.85)	107 (37.15)	**288** (**68.41)**	
Total	296 (70.31)	**125** (**29.69)**		

Variable	Urine assay method		Self‐reported method	
Age (years)	Nonadherent, *n* (%)	Adherent, *n* (%)	Nonadherent, *n* (%)	Adherent, *n* (%)
<5	33 (56.90)	25 (43.10)	44 (75.86)	14 (24.14)
5–9	102 (62.58)	61 (37.42)	116 (71.17)	47 (28.83)
10–14	121 (77.56)	35 (22.44)	104 (66.67)	52 (33.33)
>14	40 (90.91)	4 (9.09)	24 (54.55)	20 (45.45)

*Note*: Bold values are adherent rate of penicillin V prophylaxis for both urine assay (subjective) method and self‐reported method.

### 6.5 Barriers/challenges to penicillin V adherence

**Figure 3:**
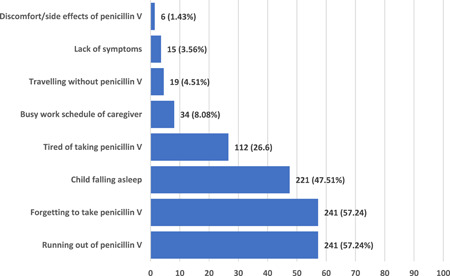
Barriers to penicillin V adherence

Multiple barriers or reasons for missing a dose of penicillin V were elicited and are presented in Figure 3. The most common barriers included forgetfulness in taking penicillin V (57.24%, *n* = 241), “running out” of penicillin V 241 (57.24%), child falling asleep (47.51%, *n* = 200), and being tired of taking penicillin V (26.60%, *n* = 112%).

### 6.6 Factors associated with both urine assay (objective) and self‐reported (subjective) method of adherence of penicillin V prophylaxis

#### 6.6.1 Urine assay (objective) method

SCD patients within the ages of 10–14 years and greater than 14 years were at a reduced odds of 0.36 (aOR = 0.36, CI = 0.17–0.80, *p* = 0.012) and 0.17 (aOR = 0.17, CI = 0.05–0.61, *p* = 0.007), respectively, to adhere to penicillin V prophylaxis. SCD patients who are cared for by their grandparents were at increased odds of 3.68 times to adhere to penicillin V (aOR = 3.68, CI = 1.03–13.15, *p* = 0.045). SCD patients cared for by married and divorced caregivers/parents were at a reduced odds of 0.32 (aOR = 0.32, CI = 0.14–0.72, *p* = 0.006) and 0.23 (aOR = 0.23, CI = 0.07–0.75, *p* = 0.014) to adhere to penicillin V, respectively. SCD patients taking homemade (herbal) medication for the treatment of SCD and those classified as taking penicillin V inappropriately were also at a reduced odds of 0.42 (aOR = 0.42, CI = 0.21–0.83, *p* = 0.013) and 0.27 (aOR = 0.27, CI = 0.11–0.67, *p* = 0.004) to adhere penicillin V, respectively.

#### 6.6.2 Self‐reported (subjective) method

SCD patients taking homemade (herbal) medication for the treatment of SCD were at a reduced odds of 0.52 to adhere to penicillin V (aOR = 0.52, CI = 0.30–0.89, *p* = 0.017). Also, SCD patients who take penicillin V inappropriately were at a reduced odds of 0.32 to adhere to penicillin V prophylaxis (aOR = 0.32, CI = 0.17–0.60, *p* < 0.000) (Table 5).

**Table 5:** Factors associated with both objective and subjective method of adherence of penicillin V prophylaxis

Variable	Penicillin medication adherence			
	Urine assay (objective) method of adherence		Self‐reported (subjective) method of adherence	
	OR (95% CI)	aOR (95% CI)	OR (95% CI)	aOR (95% CI)
Gender				
Male	Ref	Ref	Ref	Ref
Female	0.93 (0.61–1.42)	0.88 (0.55–1.41)	0.93 (0.61–1.40)	0.91 (0.58–1.42)
Age of SCD patient (years)				
<5	Ref	Ref	Ref	Ref
5–9	0.79 (0.43–1.45)	0.75 (0.36–1.55)	0.79 (0.39–1.57)	0.91 (0.41–2.03)
10–14	0.38 (0.20–0.73)**	0.36 (0.17–0.80)***	0.64 (0.32–1.27)	0.79 (0.35–1.82)
>14	0.13 (0.04–0.42)**	0.17 (0.05–0.61)**	0.38 (0.16–0.89)***	0.56 (0.20–1.52)
SCD Phenotype				
HbSC	Ref	Ref	Ref	Ref
HbSS	0.75 (0.56–1.24)	0.71 (0.40–1.26)	1.18 (0.72–1.93)	1.35 (0.77–2.35)
Residential status				
Rural	Ref	Ref	Ref	Ref
Suburban	1.43 (0.80–2.54)	1.20 (0.63–2.29)	1.29 (0.74–2.24)	1.22 (0.66–2.24)
Urban	1.03 (0.55–1.92)	0.86 (0.42–1.76)	0.89 (0.50–1.58)	0.93 (0.49–1.79)
Schooling status				
Not schooling	Ref	Ref	Ref	Ref
Schooling	1.93 (0.41–9.06)	3.49 (0.57–21.37)	0.81 (0.21–3.09)	0.76 (0.16–3.62)
Primary caregiver				
Biological parent	Ref	Ref	Ref	Ref
Grandparent	2.25 (0.97–5.26)	3.68 (1.03–13.15)***	0.99 (0.40–2.47)	1.36 (0.41–4.54)
Other	0.77 (0.27–2.15)	0.79 (0.21–2.98)	0.33 (0.13–0.79)***	0.42 (0.13–1.31)
Age of primary caregiver (years)				
<28	Ref	Ref	Ref	Ref
28–37	1.33 (0.48–3.72)	3.22 (0.80–12.91)	3.17 (1.20–8.38)***	2.53 (0.69–9.28)
38–47	0.87 (0.32–2.40)	3.00 (0.72–12.49)	1.95 (0.77–4.94)	1.65 (0.44–6.19)
>47	0.82 (0.29–2.36)	2.39 (0.51–10.93)	2.03 (0.77–5.36)	2.06 (0.51–8.39)
Marital status of caregiver				
Single	Ref	Ref	Ref	Ref
Married	0.42 (0.22–0.79)**	0.32 (0.14–0.72)**	1.09 (0.56–2.11)	0.78 (0.32–1.90)
Divorced	0.32 (0.12–0.86)***	0.23 (0.07–0.75)***	0.73 (0.29–1.81)	0.56 (0.18–1.71)
Widowed	0.46 (0.16–1.19)	0.29 (0.08–1.07)	1.21 (0.43–3.37)	1.04 (0.28–3.92)
Educational level of caregiver				
No formal education	Ref	Ref	Ref	Ref
Basic education	0.78 (0.32–1.88)	0.66 (0.25–1.78)	1.20 (0.52–2.77)	1.22 (0.49–3.03)
Secondary education	1.14 (0.44–2.95)	0.80 (0.27–2.36)	1.61 (0.64–4.05)	1.55 (0.56–4.28)
Tertiary education	1.34 (0.52–3.48)	1.06 (0.31–3.64)	1.84 (0.72–4.74)	1.30 (0.39–4.25)
Employment status of caregiver				
Unemployed	Ref	Ref	Ref	Ref
Self‐employed	0.56 (0.29–1.08)	0.72 (0.31–1.64)	1.45 (0.76–2.77)	1.49 (0.68–3.27)
Formally employed	0.61 (0.28–1.36)	0.45 (0.15–1.37)	2.4 (1.05–5.51)***	2.07 (0.68–6.27)
Mode of diagnosis of SCD patient				
NSP	Ref	Ref	Ref	Ref
Non‐NSP	0.84 (0.55–1.29)	0.78 (0.47–1.28)	1.39 (0.92–2.11)	1.23 (0.77–1.96)
Comorbidity				
No	Ref	Ref	Ref	Ref
Yes	0.25 (0.06–1.10)	0.41 (0.09–1.94)	0.44 (0.18–1.09)	0.52 (0.19–1.41)
Family history of SCD				
No	Ref	Ref	Ref	Ref
Yes	0.82 (0.52–1.28)	0.76 (0.46–1.28)	0.89 (0.58–1.37)	0.88 (0.54–1.42)
Taken homemade medication for SCD				
No	Ref	Ref	Ref	Ref
Yes	0.37 (0.20–0.69)**	0.42 (0.21–0.83)***	0.48 (0.29–0.77)**	0.52 (0.30–0.89)***
Barriers to medication adherence				
No barrier	Ref	Ref	Ref	Ref
≥1	0.42 (0.22–0.79)**	0.58 (0.27–1.24)	0.70 (0.34–1.43)	0.81 (0.34–1.83)
Intake of penicillin V				
Appropriate	Ref	Ref	Ref	Ref
Inappropriate	0.30 (0.13–0.68)**	0.27 (0.11–0.67)**	0.31 (0.18–0.56)*	0.32 (0.17–0.60)*

Abbreviations: aOR, adjusted odds ratio; CI, confidence interval; HbSC, hemoglobin SC; HbSS, hemoglobin SS; OR, crude odds ratio; Ref, reference point; SCD, sickle cell disease; SD, standard deviation.

* *p* < 0.05

** *p* < 0.01

*** *p* < 0.001.

## 7 DISCUSSION

Medication adherence in chronic disease patients is recognized as a public health problem. This is because nonadherence to medications can result in increased healthcare costs and poor health outcomes.^35^ This study used self‐reported penicillin V adherence and urine assay of penicillin V as a proxy for penicillin V adherence, and it was shown that self‐reported penicillin V adherence was higher than adherence by urinary assay. Further, there was a significant difference between the two methods. We anticipate that the disparities in penicillin V adherence between the two methods could be attributed to recall bias in reporting self‐adherence. The adherence rate observed under the objective method in the present study is relatively low compared to a study conducted in Brazil by Bitarães et al., which reported an adherence level of 56%. Differences in study design employed by both studies could contribute to the low adherence rate in the current study compared to the findings of Bitarães et al.^36^ wherein penicillin V adherence was assessed by a longitudinal method. However, our findings also corroborate with the outcome of Witherspoon and Drotar^27^ who employed a cross‐sectional design and reported an adherence level of 33.0%. The self‐reported penicillin V adherence recorded by this study was relatively high compared to a study conducted in the United States by Patel et al. where an adherence rate of 54.9% was recorded. The difference in the adherence rate may be attributed to the method of assessment used and the population that was studied. The method of assessment used in the earlier study was medication possession ratio and adherence was assessed in SCD patients with asthma.^37^ The implication of the findings vis‐à‐vis the comparison of the method offers a reliable finding in understanding the effectiveness of this treatment regimen.

Further, the observed factors contributing to penicillin V adherence which included “forgetfulness of taking penicillin V prophylaxis,” “penicillin V prophylaxis out of stock,” and “child falling asleep” are not new barriers to penicillin V adherence among children with SCD as similar findings are reported by Witherspoon and Drotar, Todd et al., and Walsh et al.^27^, ^38^, ^39^ It was further observed that the higher the observed barriers, the lower adherence recorded as shown in the bivariate analysis. It is therefore safe to say that implementing steps to address the barriers would have improved penicillin V adherence.

Adherence to penicillin V medication is crucial to improving health outcomes of SCD patients and, thus, it is important to understand the drivers of penicillin V nonadherence, especially in children. In this study, it was revealed that, the age of SCD patient and primary caregiver, marital status of the caregiver, SCD patient taking homemade medications for the treatment of SCD, and inappropriate intake of penicillin V were significantly associated with penicillin V adherence. Age is a key contributing factor in medication adherence in patients with chronic disease and this could explain our observation. According to Basheti et al.^40^ adults living with chronic conditions or increasing age is correlated with a high level of medication adherence. This is because persons who live with SCD for a long time are likely to experience the worst form of the condition which may increase the awareness of their health and so translate to adherence to their medications. However, in the case of our study, SCD children who were 10 years and more were nonadherent to penicillin V prophylaxis whilst children under 5 years were more adherent. Similar findings were reported by Teach et al. and Loiselle et al.^19^, ^41^ The reason for the observed reverse effect of living longer with SCD and improved adherence could be due to the fact that children living with chronic conditions such as SCD take responsibility for their medications as they age (usually as early as 9 years in Ghana). On the other hand, medication adherence for infant's/toddlers is determined largely by the parents/caregiver, hence such patients are most likely to administer the medication efficiently thereby enjoying the benefits associated with penicillin V prophylaxis. Another reason could be due to healthcare providers in the clinic may stress on adherence to penicillin V among children under 5 due to the high susceptible rate of infection‐related admission and complications.^19^ Also, other factors such as an individual's cognitive level, physical mobility, and self‐care abilities may also have an impact on age and adherence to medication. It is therefore important that education at the SCD clinics is heightened.

Marital status of caregiver significantly influences medication adherence levels. Our study showed that SCD children in the care of married caregivers poorly adhere to penicillin V prophylaxis than those whose caregivers are unmarried. These findings contradict the outcome of several studies.^42^, ^43^, ^44^ It was expected that the SCD children cared for by married caregivers would rather have better penicillin V adherence levels due to the perceived shared responsibility and social support it offers. Thus our finding requires further investigation. This finding may be due to the shared responsibility married caregivers will have for their child's health which may likely influence the child's adherence to medication. Poor penicillin V adherence was also observed among SCD patients cared for by divorced caregivers. This could be due to a self‐denial state of mind where the divorced caregiver is not bothered about concerns of health and life, and also a lack of financial support from a spouse. Social support intervention is recommended for widowed caregivers due to the evidence of its effectiveness in improving medication adherence as reported in some studies.^45^, ^46^, ^47^

In this present study, being cared for by grandparents was associated with a high level of adherence to penicillin V prophylaxis. Globally, grandparents play vital roles in the lives of their beloved young grandchildren. In many families, they serve as primary caregivers to their grandchildren. All the grandparents who cared for the SCD patients seen during the study were above 50 years. Evidence suggests that among adults, increasing age is associated with medication adherence.^48^, ^49^, ^50^ That is, the more one lives with a condition the more likely the person develops knowledge and awareness of the condition. Since the grandparents serve as primary caregiver for their grandchildren, they will ensure their grandchildren consume all medications which may account for the significant increase in the penicillin V adherence level in this study. Also, studies have demonstrated that grandparents are more likely to access health care services for their wards due to the strong affection they have for their grandchildren.^51^ A study conducted by Baker and Silverstein reported that grandparents who had been raising their grandchildren for at least 2 years were likely to participate in health prevention activities.^52^ Also Whitley et al.^51^ suggest that on many occassions grandparents do well in using health services on an annual basis and active engagement in receiving health care services appears to positively benefit the grandchildren.

The use of homemade (herbal) preparation was relatively common among the patients and this was significantly associated with poor adherence of SCD patients. This implies that the use of herbal preparation is not new among patients living with chronic diseases.^53^, ^54^, ^55^ Our search suggests that no study has reported on the use of herbal preparation and its influence on penicillin V consumption, we argue that the use of herbs among SCD patients is similar to other chronic conditions. Studies have revealed that the use of homemade medications is significantly associated with poorer adherence to medication among adults with other chronic conditions.^56^, ^57^ Possible inference from these observations is that patronage to herbal preparations may lead to patient withdrawal from seeking conventional treatment.^58^ Also, concurrent use of herbs and conventional medicine may expose the patients to adverse effects arising from drug interactions,^59^, ^60^, ^61^ which, in turn, decreases medication adherence. Health education messaging could be designed taking into account contextual factors such as the over‐reliant on traditional medication and its negative influence on penicillin V adherence as observed in this study.

The present study revealed inappropriate use (overuse and underuse) of penicillin V among SCD patients and this was significantly associated with poorer adherence to penicillin V among the patients in both methods of penicillin V adherence assessment. Inappropriate intake of medicines are mostly intentional among patient living with the chronic disease based on their knowledge, experience, and beliefs about the medicine.^28^ Although the study did not solicit for information on reasons for inappropriate intake of penicillin V, some reasons for inappropriate intake could be fear of potential side effects, cost of penicillin V, lack of symptoms and being worried about taking penicillin V (being dependent on penicillin V for long) which could render SCD patients taking penicillin V inappropriately.

## 8 LIMITATION

The present study has some limitations. First, patients recruited were sampled from the SPARCo registry and accessed care at the KATH SCD clinic. It, therefore, excludes SCD patients less than 2 years and also patients accessing care at other peripheral hospitals in Kumasi and other regions of Ghana. Second, this study was a cross‐sectional study where penicillin V adherence for the objective method (urine assay method) was assessed at a one‐time‐point. There is a need for future studies to assess penicillin V adherence longitudinally. Finally, the subjective nature of self‐reported adherence could introduce recall bias and hence may not give a true picture of penicillin V adherence. However, the study provides empirical evidence on adherence to penicillin V prophylaxis.

## 9 CONCLUSION

The study revealed a significant difference between the objective and subjective methods of assessment of penicillin V prophylaxis adherence. Overall, penicillin V prophylaxis adherence was relatively poor among the children with SCD in this study. Age of SCD patients, marital status of caregiver, taking of homemade medications for SCD treatment and inappropriate intake of penicillin V prophylaxis accounted for poor adherence to penicillin V prophylaxis. However, SCD children being cared for by a grandparent as primary caregiver were more adherent to penicillin V prophylaxis. The poor adherence to penicillin V prophylaxis among SCD patients is a public health concern. Therefore, it is imperative to intensify education and counseling for patients and/or caregivers about the detrimental effects of poor adherence to penicillin V prophylaxis, and adherence should be a collective responsibility of patients and caregivers.

## AUTHOR CONTRIBUTIONS

**Samuel F. Odoom**: Conceptualization; funding acquisition; investigation; methodology; project administration; writing – original draft; writing – review and editing. **Sam K. Newton**: Supervision; writing – review and editing. **Emmanuel K. Nakua**: Supervision; writing – review and editing. **Kennedy G. Boahen**: Investigation; supervision; writing – review and editing. **Samuel B. Nguah**: Conceptualization; formal analysis; supervision; writing – review and editing. **Daniel Ansong**: Supervision; writing – review and editing. **Isaac Nyanor**: Data curation; formal analysis; software; validation. **Evans X. Amuzu**: Data curation; formal analysis; project administration; software; validation. **Ernest Amanor**: Data curation; investigation; writing – review and editing. **Francis A. Osei**: Formal analysis; software; supervision; validation; writing – review and editing. **Aliyu Mohammed**: Formal analysis; supervision; writing – review and editing. **Nicholas K. Mensah**: Data curation; software; validation; writing – review and editing. **Charles Martyn‐Dickens**: Supervision; writing – review and editing. **Alex Osei‐Akoto**: Conceptualization; supervision; writing – review and editing. **Vivian Paintsil**: Conceptualization; supervision; writing – review and editing.

## CONFLICT OF INTEREST

The authors declare no conflict of interest.

## ETHICS STATEMENT

Approval for the conduct of this study was obtained from the Institutional Review Board of KATH with reference number: KATH IRB/AP/063/21. Written informed consent was obtained from caregivers and SCD patients above 7 years gave assent, respectively, after the purpose of the study was explained to participants.

## TRANSPARENCY STATEMENT

The lead author Samuel F. Odoom affirms that this manuscript is an honest, accurate, and transparent account of the study being reported; that no important aspects of the study have been omitted; and that any discrepancies from the study as planned (and, if relevant, registered) have been explained.

## Supporting information

Supporting information.

## Data Availability

The study data collected, analyzed and presented are available at the Ghana SPARCo Site and is available upon formal request.

## References

[1] WHO .Regional Office for Africa. WHO; 2020. Accessed March 13, 2015. https://www.afro.who.int/?option=com_docman%26task=doc_download%26gid=5436

[2] Davies SC , Brozovic M . The presentation, management and prophylaxis of sickle cell disease. Blood Rev. 1989;3(1):29‐44. 2650774 10.1016/0268-960x(89)90023-4

[3] Okpala I , Thomas V , Westerdale N , et al. The comprehensive care of sickle cell disease. Eur J Haematol. 2002;68(3):157‐162. 10.1034/j.1600-0609.2002.01523.x 12068796

[4] Piel FB , Hay SI , Gupta S , et al. Global burden of sickle cell anaemia in children under five, 2010‐2050: modelling based on dempgraphics, excess mortality, and interventions. PLOS Med. 2013;10 (7):e1001484. 23874164 10.1371/journal.pmed.1001484 PMC3712914

[5] Piel FB , Patil AP , Howes RE , et al. Global epidemiology of sickle haemoglobin in neonates: a contemporary geostatistical model‐based map and population estimates. Lancet. 2013;381(9861):142‐151. 23103089 10.1016/S0140-6736(12)61229-X PMC3547249

[6] Grosse SD , Odame I , Atrash HK , Amendah DD , Piel FB , Williams TN . Sickle cell disease in Africa: a neglected cause of early childhood mortality. Am J Prev Med. 2011;41:S398‐S340. 22099364 10.1016/j.amepre.2011.09.013 PMC3708126

[7] Ohene‐Frempong K , Oduro J , Tetteh H , Nkrumah F . Screening newborns for sickle cell disease in Ghana. Pediatrics. 2008;121(2):S120‐S121.

[8] Quinn CT , Rogers ZR , Buchanan GR . Survival of children with sickle cell disease. Blood. 2004;103(11):4023‐4027. 14764527 10.1182/blood-2003-11-3758 PMC1828870

[9] Koplan JP , Director M , Hughes JM , et al. Preventing pneumococcal disease among infants and young children: recommendations of the advisory committee on immunization practices (ACIP). MMWR Recomm Rep. 2000;49(RR‐9):1‐35. 11055835

[10] Davies EG , Hirst C , Lottenberg R , Dower N . Pneumococcal vaccines for sickle cell disease. Cochrane Database Syst Rev. 2004;2012(2):1‐36. 10.1002/14651858.CD003885.pub2 PMC11927627 14974042

[11] Williams TN , Uyoga S , Macharia A , et al. Bacteraemia in Kenyan children with sickle‐cell anaemia: a retrospective cohort and case‐control study. Lancet. 2009;374(9698):1364‐1370. 19747721 10.1016/S0140-6736(09)61374-X PMC2768782

[12] Ramakrishnan M , Moïsi JC , Klugman KP , et al. Increased risk of invasive bacterial infections in African people with sickle‐cell disease: a systematic review and meta‐analysis. Lancet Infect Dis. 2010;10:329‐337. 20417415 10.1016/S1473-3099(10)70055-4

[13] Gaston MH , Verter JI , Woods G , et al. Prophylaxis with oral penicillin in children with sickle cell anemia. N Engl J Med. 1986;314(25):1593‐1599. 3086721 10.1056/NEJM198606193142501

[14] Ansong D , Akoto AO , Ocloo D , Ohene‐Frempong K . Sickle cell disease: management options and challenges in developing countries. Mediterr J Hematol Infect Dis. 2013;5(1):2013062. 10.4084/MJHID.2013.062 PMC3867228 24363877

[15] World Health Organization. Adherence to Long‐Term Therapies: Evidence for Action. World Health Organization; 2003.

[16] National Institute for Health and Clinical Excellence. Medicines adherence: Involving patients in decisions about prescribed medicines and supporting adherence. NICE Guide; 2009:1‐31. https://www.nice.org.uk/guidance/cg76

[17] Winnick S , Lucas DO , Hartman AL , Toll D . How do you improve compliance? Pediatrics. 2005;115(6):e718‐e724. 15930200 10.1542/peds.2004-1133

[18] Matsui D . Current issues in pediatric medication adherence. Pediatr Drugs. 2012;9(5):283‐288. 10.2165/00148581-200709050-00001 17927300

[19] Teach SJ , Lillis KA , Grossi M . Compliance with penicillin prophylaxis in patients with sickle cell disease. Arch Pediatr Adolesc Med. 1998;152(3):274‐278. 9529466 10.1001/archpedi.152.3.274

[20] Cummins D , Heuschkel R , Davies SC . Penicillin prophylaxis in children with sickle cell disease in Brent. BMJ. 1991;302(6783):989‐990. 2039895 10.1136/bmj.302.6783.989 PMC1669289

[21] Pegelow CH , Armstrong FD , Light S , Toledano SR , Davis J . Experience with the use of prophylactic penicillin in children with sickle cell anemia. J Pediatr. 1991;118(5):736‐738. 2019928 10.1016/s0022-3476(05)80036-8

[22] Ghana Statistical Service . Ghana 2021 Population and Housing Census. 2021;1:1‐30. https://census2021.statsghana.gov.gh/

[23] Kumasi Metro . Kumasi Metropolitan Assembly, Kumasi—Government of Ghana. 2021. Accessed January 14, 2021. http://kma.gov.gh/kma/?brief-on-kma%26page=5143

[24] Ministry of Health . Komfo Anokye Teaching Hospital, Ministry of Health; 2015. Accessed July 18, 2018. http://www.moh.gov.gh/komfo-anokye-teaching-hospital/

[25] Paintsil V , Amuzu EX , Nyanor I , et al. Establishing a sickle cell disease registry in Africa: experience from the sickle Pan‐African research consortium, Kumasi–Ghana. Front Genet. 2022;13:802355. 35281803 10.3389/fgene.2022.802355 PMC8908904

[26] University of Florida. Determining Sample Size Degree of Variability. University of Florida; 2003:1‐5.

[27] Witherspoon D , Drotar D . Correlates of adherence to prophylactic penicillin therapy in children with sickle cell disease. Children's Health Care. 2006;35(4):281‐296.

[28] American Medical Association. 8 Reasons Patients Don't Take Their Medications. American Medical Association; 2020. Accessed February 27, 2022. https://www.ama-assn.org/delivering-care/patient-support-advocacy/8-reasons-patients-dont-take-their-medications

[29] Research Randomizer . 2020. Accessed July 10, 2021. https://www.randomizer.org/

[30] Nationwide Children .Clean Catch Urine Collection Guidelines for Males and Females. Nationwide Children; 2016:2‐4.

[31] Grove DC , Randall WA . Assay Methods of Antibiotics: A Laboratory Manual. Vol 96. Medical Encyclopedia. Inc; 1955:98.

[32] Dürst UN , Bruder E , Egloff L , Wüst J , Schneider J , Hirzel HO . [Micrococcus luteus: a rare pathogen of valve prosthesis endocarditis]. Z Kardiol. 1991;80(4):294‐298. 1862670

[33] Harris PA , Taylor R , Minor BL , et al. The REDCap consortium: building an international community of software platform partners. J Biomed Inf. 2019;95:103208. 10.1016/j.jbi.2019.103208 PMC7254481 31078660

[34] Bergman AB , Werner RJ . Failure of children to receive penicillin by mouth. N Engl J Med. 2010;24:1334‐1338. 10.1056/NEJM196306132682404 13970724

[35] Lam WY , Fresco P . Medication adherence measures: an overview. Biomed Res Int. 2015;2015:217047. 26539470 10.1155/2015/217047 PMC4619779

[36] Bitarães EL , Oliveira BM , Viana MB . Compliance with antibiotic prophylaxis in children with sickle cell anemia: a prospective study. J Pediatr. 2008;84(4):316‐322. 10.2223/JPED.1819 18688558

[37] Patel NG , Lindsey T , Strunk RC , DeBaun MR . Prevalence of daily medication adherence among children with sickle cell disease: a one‐year retrospective cohort analysis. Pediatr Blood Cancer. 2010;55(3):554. 20658630 10.1002/pbc.22605 PMC3665080

[38] Todd KE , Mcgrady ME , Blackmore A , Hennessey C , Luchtman‐Jones L . Assessing barriers to medication adherence in pediatric and adolescent and young adult (AYA) patients on anticoagulation. Blood. 2020;136(Suppl 1):9‐10. 10.1002/pbc.30076 36441148

[39] Walsh KE , Cutrona SL , Kavanagh PL , et al. Medication adherence among pediatric patients with sickle cell disease: a systematic review. Pediatrics. 2014;134(6):1175‐1183. 25404717 10.1542/peds.2014-0177 PMC4243064

[40] Basheti IA , Saqf El Hait S , Qunaibi EA , Aburuz S , Bulatova N . Associations between patient factors and medication adherence: a Jordanian experience. Pharm Pract. 2016;14(1):639. 10.18549/PharmPract.2016.01.639 PMC4800011 27011772

[41] Loiselle K , Lee JL , Szulczewski L , Drake S , Crosby LE , Pai ALH . Systematic and meta‐analytic review: medication adherence among pediatric patients with sickle cell disease. J Pediatr Psychol. 2016;41(4):406‐418. 26384715 10.1093/jpepsy/jsv084 PMC5896791

[42] Okoro RN , Ngong CK . Assessment of patient's antihypertensive medication adherence level in non‐comorbid hypertension in a tertiary hospital in Nigeria. Int J Biomed Sci. 2012;3(2):47‐54.

[43] Trivedi RB , Ayotte B , Edelman D , Bosworth HB . The association of emotional well‐being and marital status with treatment adherence among patients with hypertension. J Behav Med. 2008;31(6):489. 18780175 10.1007/s10865-008-9173-4 PMC3746832

[44] Adekunle WA , Olaniyan FA , Ismail WO , Adeyinka JO . Assessment of drug adherence among sickle cell disease patients attending secondary health care facility at Ibadan, south west Nigeria. Niger J Fam Pract. 2020;10(1):39‐48.

[45] Guimarães TMR , Miranda WL , Tavares MMF . O cotidiano das famílias de crianças e adolescentes portadores de anemia falciforme. Rev Bras Hematol Hemoter. 2009;31(1):9‐14.

[46] Whittemore R , Knafl K . The integrative review: updated methodology. J Adv Nurs. 2005;52(5):546‐553. 16268861 10.1111/j.1365-2648.2005.03621.x

[47] Marsh VM , Kamuya DM , Molyneux SS . “All her children are born that way”: gendered experiences of stigma in families affected by sickle cell disorder in rural Kenya. Ethn Health. 2011;16(4–5):343‐359. 21797722 10.1080/13557858.2010.541903 PMC3534410

[48] Shaya FT , Du D , Gbarayor CM , Frech‐Tamas F , Lau H , Weir MR . Predictors of compliance with antihypertensive therapy in a high‐risk medicaid population. J Natl Med Assoc. 2009;101(1):34‐39. 19245070 10.1016/s0027-9684(15)30808-7

[49] Hashmi SK , Afridi MB , Abbas K , et al. Factors associated with adherence to anti‐hypertensive treatment in Pakistan. PLoS One. 2007;2(3):e280. 17356691 10.1371/journal.pone.0000280 PMC1805684

[50] Monane M , Bohn RL , Gurwitz JH , Glynn RJ , Levin R , Avorn J . Compliance with antihypertensive therapy among elderly medicaid enrollees: the roles of age, gender, and race. Am J Public Health. 2011;86(12):1805‐1808. 10.2105/ajph.86.12.1805 PMC1380739 9003143

[51] Whitley MD , Fuller‐Thomson E , Brennestuhl S . Health characteristics of solo grandparent caregivers and single parents: a comparative profile using the behavior risk factor surveillance survey. Curr Gerontol Geriatr Res. 2015;2015:630717. 26448744 10.1155/2015/630717 PMC4581507

[52] Baker LA , Silverstein M . Preventive health behaviors among grandmothers raising grandchildren. J Gerontol B Psychol Sci Soc Sci. 2008;63(5):S304‐S311. 18818451 10.1093/geronb/63.5.s304 PMC2633920

[53] Lubega M , Osingada CP , Kasirye P . Use of herbal medicine by caregivers in the management of children with sickle cell disease in Mulago National Referral Hospital—Uganda. Pan Afr Med J. 2021;39:163. 34548892 10.11604/pamj.2021.39.163.20740 PMC8435373

[54] Busari AA , Mufutau MA . High prevalence of complementary and alternative medicine use among patients with sickle cell disease in a tertiary hospital in Lagos, South West, Nigeria. BMC Complement Altern Med. 2017;17(1):299. 28592256 10.1186/s12906-017-1812-2 PMC5463406

[55] Majumdar S , Thompson W , Ahmad N , Gordon C , Addison C . The use and effectiveness of complementary and alternative medicine for pain in sickle cell anemia. Complement Ther Clin Pract. 2013;19(4):184‐187. 24199970 10.1016/j.ctcp.2013.05.003

[56] Açıkgöz SK , Açıkgöz E , Topal S , et al. Effect of herbal medicine use on medication adherence of cardiology patients. Complement Ther Med. 2014;22(4):648‐654. 25146069 10.1016/j.ctim.2014.05.013

[57] Islamoglu MS , Borku Uysal B , Yavuzer S , Cengiz M . Does the use of herbal medicine affect adherence to medication—a cross sectional study of outpatients with chronic disease? Eur J Integr Med. 2021;44:101326.

[58] Atwine F , Hultsjö S , Albin B , Hjelm K . Health‐care seeking behaviour and the use of traditional medicine among persons with type 2 diabetes in south‐western Uganda: a study of focus group interviews. Pan Afr Med J. 2015;20:76. 26090034 10.11604/pamj.2015.20.76.5497 PMC4450037

[59] Ameade EPK , Ibrahim M , Ibrahim HS , Habib RH , Gbedema SY . Concurrent use of herbal and orthodox medicines among residents of Tamale, Northern Ghana, Who Patronize Hospitals and Herbal Clinics. Evid Based Complement Alternat Med. 2018;2018:1289125. 29743917 10.1155/2018/1289125 PMC5884405

[60] Izzo AA . Interactions between herbs and conventional drugs: overview of the clinical data. Med Princ Pract. 2012;21(5):404‐428. 22236736 10.1159/000334488

[61] Lim JW , Chee SX , Wong WJ , He QL , Lau TC . Traditional Chinese medicine: herb‐drug interactions with aspirin. Singapore Med J. 2018;59(5):230‐239. 29796686 10.11622/smedj.2018051 PMC5966631

