# Supplementary material for: Penicillin V prophylaxis uptake among children living with sickle cell disease in a specialist sickle cell clinic in Ghana: A cross‐sectional study
Source: Health Sci Rep. 2022 Nov 24;5(6):e953. doi: 10.1002/hsr2.953 (PMC9686354; doi:10.1002/hsr2.953)
Supplement: Supplementary file 1 — Supporting information. [file HSR2-5-e953-s001.docx]

**Metadata**

|  | Study ID |  |
| --- | --- | --- |
|  | Date of interview (DD/MM/YYYY) |  |
|  | Hospital Record ID |  |
|  | Initials of SCD patients |  |
|  | Person completing the questionnaire | ○ Caregiver/parents  ○ Both caregiver and patient |

**Section A: *Background Characteristics of SCD patients*** **and Primary Caregiver/Parents**

| **Q#** | **Question Item** | **Response** |
| --- | --- | --- |
| **BC1** | Age | ○ Month  ○ Years |
| **BC1a** | Age ***(completed months)*** |  |
| **BC1b** | Age ***(completed years)*** |  |
| **BC2** | Gender of patient | ○ Male  ○ Female |
| **BC3** | Haemoglobinopathy (Sickle Cell Disease phenotype) | ○ HbSS  ○ HbSC  ○ HbSβ^+^thalasemia  ○ HbSβ^0^thalasemia |
| **BC4** | Residential status | ○ Rural  ○ Suburban  ○ Urban |
| **BC5** | Is SCD patient schooling? | ○ No  ○ Yes |
| **BC5a** | Educational level of SCD patient | ○ Pre-school  ○ Primary  ○ JHS  ○ SHS |
| **BC6** | Primary caregiver | ○ Biological parent  ○ Grandparent  ○ Other (specify)…………………………….. |
| ***Background Characteristics of Primary Caregivers*** | | |
| **BC7** | Age of primary caregiver/parent |  |
| **BC8** | Family size |  |
| **BC9** | Educational level of caregiver/parent | ○ No formal education  ○ Primary  ○ JHS  ○ SHS  ○ Tertiary |
| **BC10** | Employment status of caregiver/parent | ○ Unemployed  ○ Self-employed  ○ Formally employed |
| **BC11** | Marital status of caregiver/parent | ○ Single  ○ Cohabitation  ○ Married  ○ Divorced  ○ Widowed |

**Section B: Clinical History of SCD Patients**

| **Q#** | **Question Item** | **Response** |
| --- | --- | --- |
| **CH1** | **Mode of diagnosis of SCD** | ○ Non-NSP  ○ NSP |
| **C2** | How long have you been accessing medical services in the KATH paediatric SCD clinic | ○ Months (specify)…………………….  ○ Years (specify)…………………………… |
| **CH3** | Have you been diagnosed of any medical condition apart from SCD? | ○ No  ○ Yes |
| **CH3a** | Indicate the known medical condition | ○ Congenital Heart Disease  ○ Cerebral Palsy  ○ Pulmonary TB  ○ Retroviral Infection  ○ Birth Asphyxia  ○ Other (specify)…………………………………….. |
| **CH4** | Do you have a relative with SCD? | ○ No  ○ Yes |
| **CH4a** | Which of your relatives have SCD? | ○ Mother  ○ Father  ○ Auntie/cousin  ○ Siblings  ○ Grandparent  ○ Other (specify)…………………………………… |
| **CH5** | Has your child ever taken any home-made SCD medication? | ○ No  ○ Yes |
| **CH6** | How many times a day do you take oral penicillin? | ○ 1 time  ○ 2 times  ○ 3 times  ○ 4 times |
| **CH7** | Has your child ever taken any home-made SCD medication? | ○ No  ○ Yes |

**Section C:** **Medication (Penicillin V) Adherence & General Barriers to Penicillin Adherence**

| ***Micrococcus luteus* Disk Diffusion Method of Penicillin Adherence** | | **Yes** | **No** |
| --- | --- | --- | --- |
| **MA9** | Is there any evidence of zone of inhibited bacterial growth around the disk? ***[Laboratory confirmation]*** |  |  |
| **Self-reported (Subjective) Method of Adherence** | | | |
| **MA10** | Did you/your child take his/her penicillin V medication yesterday night? |  |  |
| **MA10a** | At what time did you/your child take the penicillin V medication yesterday night? ***(HH:MM)*** |  | |
| **Barriers to Penicillin Adherence** | | | |
| There are many barriers to penicillin medications reported by other patients living with SCD in Ghana and other countries. We want to find out what are some of the barriers that hinders your uptake of penicillin? | | | |
| **MA11** | Indicate the barrier(s) to penicillin adherence you have encountered as at the time you started taking penicillin ***[Tick all that apply]*** | □ Discomfort/side effects of penicillin V  □ Lack of symptoms  □ Travelling without penicillin V  □ Busy work schedule  □ Tired of taking penicillin V  □ Forgetting to take penicillin V to take the medicine  □ Running out of penicillin V  □ Child falling asleep  □ Other (specify)……………… | |
